# An exploratory, qualitative study of treatment preferences among participants in a TB therapeutic trial

**DOI:** 10.5588/ijtldopen.26.0179

**Published:** 2026-07-13

**Authors:** F. Mugodhi, C. Kanyama, F. Makiya, J. Metcalfe, C. Potani, K.K. Scarsi, I. Weir, J. Furin, on behalf of the A5362/CLO-FAST study team

**Affiliations:** 1 University of Zimbabwe-Clinical Trials Research Centre (UZ-CTRC), Harare, Zimbabwe;; 2 University of North Carolina Project, Lilongwe, Malawi;; 3 Johns Hopkins Project, Blantyre Clinical Research Site, Blantyre, Malawi;; 4 Division of Pulmonary and Critical Care Medicine, San Francisco General Hospital and Trauma Center, University of California, San Francisco (UCSF), CA, USA;; 5 College of Pharmacy, University of Nebraska Medical Center, Omaha, NE, USA;; 6 Center for Biostatistics in AIDS Research in the Department of Biostatistics, Harvard T.H. Chan School of Public Health, Boston, MA, USA;; 7 Department of Global Health and Social Medicine, Harvard Medical School, Boston, MA, USA.

**Keywords:** tuberculosis, clinical trial, clofazimine, stigma, hyperpigmentation, preferences, NCT04311502

## Abstract

**BACKGROUND:**

TB is a leading cause of morbidity and mortality, and TB treatment shortening is a focus of TB therapeutic trials. Clofazimine (CFZ) has been proposed as part of a shorter TB regimen but the drug causes skin hyperpigmentation. Patient preferences around shorter regimens with CFZ should be documented.

**METHODS:**

We performed an exploratory, qualitative sub-study using in-depth interviews among purposively selected participants in the CLO-FAST trial (NCT04311502). Participants were asked about their experience with their assigned treatment regimen and about preferences for treatment. Data were analysed by trained qualitative researchers using the theoretical framework of acceptability.

**RESULTS:**

Twenty-three participants from India, Malawi, and Zimbabwe were selected from 89 participants randomised to two study arms in the parent trial. Most participants expressed preference for shorter regimen even if it led to skin hyperpigmentation. Preferences for a longer regimen may have been influenced by anticipated concerns around inadvertent disclosure and by a belief that longer regimens were more potent.

**CONCLUSION:**

Preference assessment can be successfully built into therapeutic TB trials. Most participants preferred shorter treatment but concerns about potency and skin discolouration were expressed. Additional work is needed to describe therapeutic preferences among people with TB.

Each year, 10 million people around the world become newly sick with TB.^1^ Treating TB is challenging, as multi-month courses of four to seven medications must be taken daily to achieve non-relapsing cure.^2^ Despite being a treatable and preventable infection, TB kills more than 1.2 million people globally every year.^3^ The past decade has witnessed notable achievements in TB therapeutics, with new drugs developed or repurposed to target *Mycobacterium tuberculosis*.^4^ One of these agents is the riminophenazine agent clofazimine (CFZ), which has been used to treat leprosy and drug-resistant TB for decades. Preclinical studies suggest using CFZ within first-line drug-susceptible TB (DS-TB) treatment regimens could allow for significantly shortened treatment duration, making it more tolerable for people diagnosed with the disease.^5^ Unfortunately, CFZ is associated with skin hyperpigmentation which can persist for weeks to months after therapy has been completed.^6^ Because TB is a highly stigmatised condition,^7^ drugs that cause a change in appearance could have serious consequences for individuals who receive them, especially if they lead to inadvertent disclosure.^8^

It is unknown how acceptable a shorter regimen containing CFZ might be to people being offered such treatment. We thus sought to explore the acceptability of such a regimen using qualitative methods^9^ as part of a multi-centre trial assessing the efficacy and safety of a shorter, CFZ-containing regimen for DS-TB.

## METHODS

This was an exploratory qualitative sub-study using in-depth interviews (IDIs) among a subset of participants selected from the CLO-FAST trial (NCT04311502, registered 5 November 2021).^10^ The Advancing Clinical Therapeutics Globally (ACTG) network launched the A5362 CLO-FAST trial in 2020. This phase 2C trial compared the 26-week standard of care regimen consisting of isoniazid, rifampicin, pyrazinamide, and ethambutol (standard of care or ‘SOC’ arm) with a 13-week regimen consisting of CFZ, rifapentine, isoniazid, pyrazinamide, and ethambutol (‘CFZ arm). The study had an open-label design, and all participants were provided with explicit details about all of the possible regimens to which they might be assigned, including the duration of therapy and the potential adverse events associated with the drugs, including a possible change in skin pigmentation.

Participants at three countries (India, two sites in Malawi, and Zimbabwe) were purposively selected based on site, gender, and age to participate in an exploratory IDI designed to assess their experience with the treatment regimen they received. Interviews were conducted by trained study staff after at least four weeks of TB treatment had been administered. Study staff explained the purpose of the interview before beginning the discussion. All study staff who were conducting the interviews underwent supplemental training in qualitative methods and open-ended interviewing techniques. This included training on the specific questions being asked in this study, the use of interview probes to further explore issues raised by the study participants, providing feedback to participants to ensure their responses were understood, complete, and well documented, and techniques for capturing/recording the participant responses. This supplemental training was conducted by a trained qualitative researcher (JF) with 30 years of experience carrying our open-ended interviews among people living with TB.

The questions asked during the interviews were developed from pilot interview done by one of the authors (JF) with four key informants who had received CFZ during TB treatment. The theoretical model guiding question development was the Theoretical Framework of Acceptability (TFA). The questions were initially developed in English. They were then translated and back translated into the local language of the participants by trained study staff who were fluent in those languages. The questions were piloted by local staff using standard ACTG operating procedures. Responses were recorded in the primary language of the participant then translated into English for analysis. Responses were captured with verbatim quotations in a case report form by trained site investigators and analysed at the end of the trial. Interviews were checked by a researcher trained in qualitative methods at each site to ensure completeness and accuracy at the time of the interview. Interview completeness was further assessed at the end of the study by one of the central qualitative researchers (JF) who then communicated queries about completeness and accuracy with the researcher trained in qualitative methods at each site. Responses to the queries were then checked with the primary interviewer, and, where necessary, with the respondent and communicated back to the central qualitative research team. The interview questions are included in the Supplementary Data. Due to budgetary limitations and the exploratory nature of the study, the interviews were not recorded. Interviews were conducted at the participating clinical research sites.

Standard qualitative methods were used to analyse the responses for themes and content.^9^ The interview responses were coded by two experienced qualitative researchers employed by the ACTG (JF developed the initial codes which were then reviewed by F. M.) and then shared with the (sub) study team for further input. The theoretical model used in the analysis was the TFA developed for paediatric TB.^11^ The TFA focuses on domains that make up most factors which are relevant to the overall acceptability of TB treatment, which are usability, receptivity, and integration and which are described in more detail in the Supplementary Data. The intended sample size was 40 participants, but this was not reached as the parent protocol was halted early by the protocol’s Data and Safety Monitoring Board. Data saturation was thus not considered in the analysis of the sub-study.

Researchers engaged in reflexive practice to reduce bias and maintain objectivity. Whenever possible, the analysis team tried to account for these biases and limitations, which are further detailed in the limitations section of this paper. Participants did not review the study data, but findings from the study were shared with them following usual ACTG practices.

### Ethical statement

The trial was registered with ClinicalTrials.gov: NCT04311502. A central institutional review board and national and local ethics committees approved the protocol. The ACTG Global Community Advisory Board as well as local community advisory boards at recruiting sites provided input into, and approved, the protocol. All methods/study procedures were carried out in accordance with the revised Declaration of Helsinki 2013 and 2024.

## RESULTS

Twenty-three participants from India, Malawi, and Zimbabwe were selected from the 89 participants randomised to the two study arms in the parent trial. All 23 gave consent and participated in the IDIs. Demographics are included in the Table. A large number of the participants were between the ages of 26–35 while 69.6 % were male. The treatment arms were almost equally divided, with 12 participants in the CFZ arm and 11 in the SOC arm.

**Table.** Participant demographics.

Variable	N (%)
Age	Range: 21–56 years
	21–25: 2 (8.7%)
	26–35: 11 (47.8%)
	36–45: 8 (34.8%)
	46 and above: 2 (8.7%)
Gender	F: 7 (30.4%)
	M: 16 (69.6%)
Treatment arm	3-month clofazimine: 12 (52.2%)
	6-month standard of care: 11 (47.8%)
Country	India: 6 (26.1%)
	Malawi: 11 (47.8%)
	Zimbabwe: 6 (26.1%)

Of the participants, 22 were interviewed once and 1 participant was interviewed twice when she decided to leave the trial early in order to capture any significant changes to her earlier responses. Thus, a total of 24 interviews were completed. However, because her responses were the same in the two interviews, only one set of interview responses contributed to the results.

### Treatment regimen preferences

Almost two thirds of the participants reported that they would prefer a shorter (3-month) regimen that caused skin changes compared with a 6-month regimen that did not cause skin changes. Some illustrative quotes from participants are included below:

I [would]choose treatment for 3 months with my skin changing complexion because taking pills for 6 months is painful. Just being a person always on pills for a long time ends up not being ideal, it’s worrisome always taking pills.

(Male aged 39, CFZ arm)

I would choose treatment for 3 months even though the skin would change because what would be important to me is the effectiveness of the drugs and getting better quickly.

(Female aged 27, CFZ arm)

It is important that a ‘shorter’ regimen was not always seen as a better regimen, and almost one third of the participants expressed a preference for a longer regimen. As one participant reported:

I would choose the 6 months because I do not want any changes on my skin or appearance because even after treatment, the changes may still remain. This may affect you even where you go anywhere or look for a job.

(Male aged 29, SOC arm)

And another stated:

I would prefer the 6-month treatment because the skin changes would make people question my health status. This would in turn probably lead to rejection and stigmatization from people I mix with in the community or at my workplace.

(Male aged 32, SOC arm)

While most who expressed preference for a longer regimen did so due to fears about skin colour changes and fear of stigmatisation, some participants felt a longer regimen would be more likely to cure them or would be more potent against TB, as illustrated below:

I feel the longer treatment is better than the shorter treatment since shorter can be inadequate then and chances [are] that some infection still remains. I feel 6 months will cure completely and I will get cured too.

(Male aged 56, SOC arm)

Some expressed no preference and said they would take any regimen that would cure them, as noted below:

I would have chosen any treatment that would heal me. What I want is healing.

(Male aged 33, CFZ arm)

Importantly, most of the people who reported that they would prefer a longer regimen did not actually receive CFZ (i.e., they were assigned to the SOC arm). This suggests an element of anticipated stigma^12^ which might have been mitigated by lived/actual experiences with skin colour changes. This can be seen in the quotation below from a participant in the CFZ arm:

Three months treatment is very good because I can get well soon and start living with my children. Even if my colour changes my priority is to get well soon. I feel very good about the fact that I can get well soon and keep my children closer to me.

(Female aged 29, CFZ arm)

Participants also expressed a preference for the regimen that they actually received, since they felt it was ‘working’ for them. This is seen in the participant quote below:

I would choose the 6 months regimen because it has helped me a lot and I have not experienced anything bad.

(Male aged 38, SOC arm)

## DISCUSSION

This was a qualitative, exploratory sub-study of the A5362 trial which aimed to elicit participants’ preferences regarding a shorter TB treatment regimen that contained the drug CFZ which causes skin hyperpigmentation. The main finding of our exploratory sub-study was that while most people reported preferring shorter regimens even if they led to skin colour changes, some participants reported preferring a longer regimen due to their fears of inadvertent disclosure. This appeared to be more common among participants who did not receive CFZ and may reflect the importance of anticipated stigma as a plausible explanation in driving treatment preferences, although this preliminary finding needs to be confirmed with future, more rigorous studies. The sub-study demonstrated the feasibility of including qualitative research within larger randomised TB therapeutic trials.

The sub-study revealed a split in treatment regimen preferences among participants. Some preferred a shorter, more intensive regimen despite the risk of side effects, while others favoured a longer regimen to avoid visible changes in appearance and because they felt it was more effective. This finding highlights the need to balance medical efficacy with the potential for social repercussions in TB treatment. Participants who preferred the shorter regimen often cited the desire to return to their normal roles more quickly and reduce the daily burden of taking multiple pills. This preference for a shorter treatment duration aligns with the findings of Wademan et al.,^13^ who reported that caregivers preferred dispersible tablet formulations for children due to their ease of use and shorter treatment duration. On the other hand, participants who preferred the longer regimen were concerned about the visible side effects of CFZ, such as skin discolouration. Data from our sub-study reveal that some participants who preferred the longer regimen were driven to this choice by a fear of stigma: those who reported preferring a longer regimen to avoid skin changes did not actually take the regimen with CFZ. This concern reflects an anticipatory stigma, where individuals fear negative social consequences even if they have not yet experienced them. Stadler et al.^14^ noted that while shorter regimens are appealing, the side effects can significantly influence patient preference and perceived treatment acceptability. Our findings suggest that while some patients prioritise the convenience and potential efficacy of shorter regimens, others are more concerned about the social implications of visible side effects, highlighting the need for personalised treatment approaches. Finally, we note that this was an open-label trial and participants were aware of the regimen they were receiving. This could have had an impact on their stated treatment preferences, as they may have simply been stating they preferred the regimen they actually received.

This sub-study had several limitations that may have impacted its findings and generalisability. Firstly, the research was conducted across only three sites, limiting the diversity of the sample and potentially overlooking regional variations in TB treatment experiences and social contexts. Secondly, this was an exploratory study meant to demonstrate the feasibility of asking about treatment preferences within a TB therapeutic trial. The lack of audio recording and the absence of supplemental field notes was the major limitation of this study. Without audio recordings, researchers relied on written notes and memory, which might not have captured the full depth and nuance of participants’ responses. This limitation could have affected the richness and accuracy of the qualitative data. Furthermore, the lack of recorded interviews could have introduced multiple other biases, including selective capture and interpretation bias. We tried to minimise these risks through the use of experienced and trained interviewers who underwent supplemental training, but it must be acknowledged that more rigorous research is needed to confirm the preliminary findings we report here. Lastly, the A5362 trial was halted earlier than planned by the Data and Safety Monitoring Board although neither interviewers nor participants were aware of this decision during data collection. The early termination of the A5362 trial resulted in a smaller sample size and possibly limited the exploration of themes that might have emerged with a larger and more diverse participant pool. Despite these constraints, the findings provide valuable insights into TB treatment preferences, the possible impact of anticipated stigma on treatment preferences, and the social dynamics of disclosure, highlighting areas for further research and intervention.

## CONCLUSION

People with TB enrolled in A5362 expressed different preferences regarding their treatment. Most people reported that they would prefer a shorter regimen lasting 3 months compared with a longer regimen lasting 6 months. Although therapeutic duration was an important factor in determining what participants preferred, anticipated stigma due to inadvertent disclosure and concerns about regimen potency were also reported by participants as reasons for preferring a longer treatment. These findings are preliminary and need to be confirmed in more rigorous studies on TB treatment preferences. Qualitative research documenting the experiences of people on TB treatment can and should be embedded in TB therapeutic trials to better describe the experiences of people enrolled in such research. Such data could provide important insights to support programmatic use of novel TB regimens.

## Supplementary Material
