# Supplementary material for: An exploratory, qualitative study of treatment preferences among participants in a TB therapeutic trial
Source: IJTLD Open. 2026 Jul 13;3(7):466–70. doi: 10.5588/ijtldopen.26.0179 (PMC13362207; doi:10.5588/ijtldopen.26.0179)
Supplement: Supplementary file 1 [file ijtldopen26-0179_supplementarydata1.pdf]

Supplementary Annex

Annex 1: Open-ended interview questions:

1. Who have you told that you have been diagnosed with tuberculosis? Why did you tell these people?
2. Do you worry about people you did not tell that you have been diagnosed with TB finding out that you have TB? Why? Are there any people you are most worried might find out you have TB?
3. Is there anything about the treatment you have been taking that you worry might identify you as someone who has TB? If so, please describe:
4. What do you think might happen to you if people you did not tell you have tuberculosis find out that you have the disease?
5. Has anything bad ever happened to you when people have found out you have tuberculosis? If yes, please describe
6. If you had to choose between taking medicines for TB for 6 months with a treatment that caused no changes in your appearance OR taking a treatment for months that might cause your skin to change, which would you prefer? Why?
7. Is there anything else you would like to tell us about your experience being diagnosed and treated for TB?

Annex 2: Theoretical Framework of Acceptability (from Wademan DT, Palmer M, Purchase S, van der Zalm MM, Osman M, Garcia-Prats AJ, et al. (2022) Toward a conceptual framework of the acceptability of tuberculosis treatment in children using a theory generative approach. PLOS Glob Public Health 2(12): e0001267. https://doi.org/10.1371/journal.pgph.0001267)

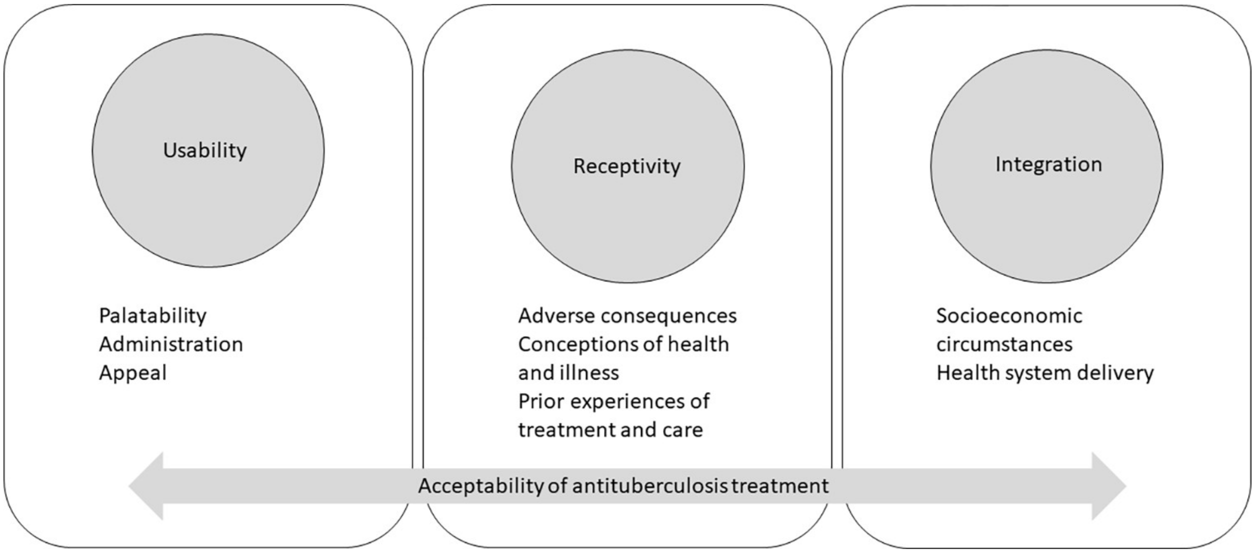

Annex 3: Protocol Team Members

Appendix

<u>Protocol Team Members</u>

<u>Chair</u>

John Metcalfe, MD, PhD, MPH
University of California, San Francisco Division of Pulmonary/Critical Care Med
San Francisco General Hospital
1001 Potrero Avenue, Room 5K1
San Francisco, CA 94110
Phone: 415-206-4679 or 415-596-9348
E-mail: john.metcalfe@ucsf.edu

<u>Co-Vice Chairs</u>

Samuel Pierre, MD
Les Centres Gheskio CRS
83 Boulevard Harry Truman
Port-au-Prince, HT-6110
HAITI

Phone: 509-3740-7711
E-mail: pierresamuel1@yahoo.fr

Kimberly Scarsi, PharmD, MS
University of Nebraska Medical Center
Department of Pharmacy Practice, Rm 3021
986145 Nebraska Medical Center
Omaha, NE 68198-6145
Phone: 402-559-9916
E-mail: kim.scarsi@unmc.edu

DAIDS Clinical Representatives

Richard Hafner, MD
Chief, TB Clinical Research Branch
DAIDS/NIAID/NIH/TBCB
5601 Fishers Lane, Room 9E30
Rockville, MD 20852
Phone: 301-435-3766
E-mail: rhafner@niaid.nih.gov

Melanie Michele Goth, MD, FAAP
TB Clinical Research Branch TRP/DAIDS/NIAID
5601 Fishers Lane
Room 9E36
MSC 9829 Rockville, MD 20892-9829
Phone 301-761-7490
E-mail: melanie.goth@nih.gov

Clinical Trials Specialist

Austin Leah Van Grack, MPH
ACTG Network Coordinating Center
Social & Scientific Systems, Inc.,
A DLH Holdings Company
8757 Georgia Avenue, 12th Floor
Silver Spring, MD 20910
Phone: 301-628-3033
E-mail: austin.vangrack@dlhcorp.com

Statisticians

Jorge Tomas Leon-Cruz
Statistical & Data Analysis Center
Harvard School of Public Health
FXB building, Room 549
Boston, MA 02115

Phone: 617-432-7469
E-mail: jleoncru@sdac.harvard.edu

Isabelle R. Weir, PhD, MA
Statistical & Data Analysis Center
Harvard School of Public Health
651 Huntington Avenue
Boston, MA 02115
Phone: 617-432-2998
E-mail: iweir@sdac.harvard.edu

Data Managers

Scott Anderson, MS
Frontier Science &
Technology Research
Foundation, Inc. 4033 Maple Road
Amherst, NY 14226
Phone: 716-834-0900
E-mail:
sanders@frontierscience.org

Jenna Meldrum, BA
Frontier Science
Foundation 4033
Maple Road
Amherst, NY 14226
Phone: 716-834-0900
E-mail:
meldrum@frontierscience.org

Brooke Altman
Frontier Science Foundation
4033 Maple Road
Amherst, NY 14226
Phone: 716-834-0900
E-mail: altman@frontierscience.org

DAIDS Pharmacist

Justine Beck, PharmD, BCPS, RPh
Pharmaceutical Affairs Branch
OCSO/DAIDS/NIAID/NIH/DHHS
5601 Fishers Lane, 9D39, MSC 9829
Rockville, MD 20852

Phone: 301-761-5288
E-mail: justine.beck@nih.gov

Pharmacologist
Gary Maartens, MBChB, MMed
University of Cape Town
Division of Clinical Pharmacology
Anzio Road, Cape Town 7925
SOUTH AFRICA
Phone: 27-21-4066286
E-mail: gary.maartens@uct.ac.za

Pharmacometrician
Elin Svensson, PhD
Radboud University Medical Center
Department of Pharmacy
P.O. Box 9101, 62 HB Nijmegen THE NETHERLANDS
Phone: 31-623217888
E-mail: elin.svensson@radboudumc.nl

Dermatologist
Maria Wei, MD, PhD
Department of Dermatology
VA 190, 4150 Clement Street
San Francisco, CA 94121
E-mail: maria.wei@ucsf.edu

Investigators
Richard E. Chaisson, MD
Division of Infectious Diseases
Johns Hopkins University
1503 E. Jefferson Street
Baltimore, MD 21231
Phone: 410-955-1755
E-mail: rchaiss@jhmi.edu

Jennifer Furin, MD, PhD
Case CRS,
2120 Circle Drive, Room E-202
Cleveland, OH 44106
Phone: 216-368-6727
E-mail: jjf38@case.edu

Jacques Grosset, MD

Johns Hopkins University CRS
CRB2, Room 105
1550 Orleans Street
Baltimore, MD 21231-1044
Phone: 410-502-8234
E-mail:jgrosse4@gmail.com

David W. Haas, MD
Vanderbilt Therapeutics CRS
One Hundred Oaks
719 Thompson Lane, Suite 47183
Nashville, TN 37204
Phone: 615-936-8594
E-mail: david.haas@vanderbilt.edu

Natthapol Kosashunhanan, MD
Chiang Mai University HIV Treatment CRS
Chiang Mai University Research Institute for
Health Sciences
110 Intavaroros Road
Chiang Mai 50200
THAILAND
Phone: 66-53-3945055 Ext. 354
E-mail: natapolk@yahoo.com

Rahul Lokande, MD
Byramjee Jeejeebhoy Medical College (BJMC)
CRS (31441)
Pulmonary Medicine Department OPD
NO. 34, Sassoon General Hospital
Jai Prakash Narayan Road
Pune 411001
INDIA
Phone: 91-98-22894972
E-mail: drrahul 2007@yahoo.com

Alberto Mendoza-Ticona MD,
Socios en Salud Sucursal Peru CRS
(31985) Jr. Puno 279, Cercado de Lima.
Lima 15001
PERU
Phone: 51 953982909
E-mail: amendoza ses@pih.org

Gus Rosania, PhD
Department of Pharmaceutical Sciences
University of Michigan College of Pharmacy
428 Church Street
Ann Arbor, MI 48109
Phone: 734-358-5661
E-mail: grosania@med.umich.edu

Wadzanai Samaneka MBChB, MSc
Milton Park CRS
31 Lawson Avenue, Milton Park
Harare 263
ZIMBABWE
Phone: 263-242-705986
E-mail: samaneka@uz-ctrc.org

International Program Specialist

Akbar Shahkolahi, PhD
ACTG Network Coordinating Center
Social and Scientific Systems, Inc.,
A DLH Holdings Company
8757 Georgia Avenue, 12th Floor
Silver Spring, MD 20910
Phone: 301-628-3318
E-mail: akbar.shahkolahi@dlhcorp.com

Field Representative

Joan Coetzee, CPN, PN
Tygerberg Hospital
Ward J8/ Francie van Zijl Drive
Parow Valley, 7505
Cape Town 7505
SOUTH AFRICA
Phone: 27-21-9384157
E-mail: joan@sun.ac.za

Community Scientific Subcommittee (CSS)
Representative

Anne Wambui Njoroge
Moi University Clinical Research Center (MUCRC)
CRS (12601)
Chandaria Cancer and Chronic Diseases Centre at Moi Teaching and Referral Hospital
Nandi Road, P.O. Box: 4606
Eldoret 30100

KENYA
Phone: 254-728934777
E-mail: anbartilol@yahoo.com

Laboratory Data Managers

Brooke Altman
Frontier Science & Technology Research Foundation
4033 Maple Road
Amherst, NY 14226
Phone: 716-834-0900
E-mail: altman@frontier-science.org

Laura Hovind, BS, MS
Frontier Science & Technology Research
Foundation
4033 Maple Road
Amherst, NY 14226
Phone: 716-834-0900 x7468
E-mail: hovind@fstrf.org

Kevin Knowles
Frontier Science & Technology Research
Foundation
4033 Maple Road
Amherst, NY 14226
Phone: 716-834-0900 x7238
E-mail: knowles@frontierscience.org

Laboratory Technologists

Brian Clagett, BA
Case CRS
BRB, Room 1048B
2109 Adelbert Road
Cleveland, OH 44106
Phone: 216-368-4853
E-mail: bmc@case.edu

Neeta N. Pradhan, MSC
Byramjee Jeejeebhoy Government
Medical College CRS (31441)
Jaiprakash Narayan Road
P.O. Box 411001
Pune 411001
INDIA

Phone: 985-0044897
E-mail: neetapradhanpune@gmail.com

Laboratory Specialists

Afton Dorasamy, BS
ACTG Laboratory Center
University of California Los Angeles
11075 Santa Monica Blvd. Suite #200
Los Angeles, CA 90025
Phone: 443-972-0685
E-mail: adorasamy@milabcentral.org

Cherisse Heirs, BA
ACTG Laboratory Center
University of California Los Angeles
11075 Santa Monica Blvd. Suite #200
Los Angeles, CA 90025
Phone: 585-397-4730
Email: cheirs@milabcentral.org

Source Document Specialist

Josie Marshall, BS
ACTG Network Coordinating Center
Social and Scientific Systems, inc.,
A DLH Holdings Company
8757 Georgia Avenue, 12th Floor
Silver Spring, MD 20910
Phone: 303-724-0803
E-mail: josie.marshall@dlhcorp.com
